# Case Report: An “Immune-Cold” EGFR Mutant NSCLC With Strong PD-L1 Expression Shows Resistance to Chemo-Immunotherapy

**DOI:** 10.3389/fonc.2022.765997

**Published:** 2022-02-22

**Authors:** Qian Zhao, Xue Zhang, Qiang Ma, Nuo Luo, Zhulin Liu, Renyuan Wang, Yong He, Li Li

**Affiliations:** ^1^ Department of Respiratory Disease, Daping Hospital, Third Military Medical University (Army Medical University), Chongqing, China; ^2^ Department of Pathology, Daping Hospital, Third Military Medical University (Army Medical University), Chongqing, China

**Keywords:** chemo-immunotherapy, EGFR, case report, tumor microenvironment, resistance

## Abstract

Long-term survival benefit has been noticed in non-small-cell lung cancer (NSCLC) patients treated with immune checkpoint inhibitors (ICIs), such as PD-1 inhibitors. However, it is still controversial whether patients with EGFR-activating mutations may benefit from ICIs. Recently, in stage IIIA NSCLC, chemo-immunotherapy has led to significant pathological response, yet patients with the presence of known EGFR mutations were excluded from some randomized trials of neoadjuvant therapy. Herein, we report a case of a 50-year-old female patient, who was initially diagnosed as stage IIIA lung squamous cell carcinoma. Immunohistochemistry analysis showed that the patient presented with high PD-L1 expression. Then, chemo-immunotherapy was given to the patient but the disease progressed quickly with distant metastasis. A re-biopsy revealed a poorly differentiated lung adenocarcinoma together with EGFR p.L858R mutation. Then the patient received gefitinib, which resulted in significant regression of primary lung lesion. A detailed examination of pre-treatment tumor sections demonstrated rare infiltration of CD8^+^ T cells, indicating that the current patient presented with an “immune-cold” microenvironment, which might explain the primary resistance to chemo-immunotherapy. Taken together, our case indicated that comprehensive detection of PD-L1 expression, driver gene status, together with tumor immune microenvironment, may offer a better prediction of treatment efficacy.

## Introduction

Immune checkpoint inhibitors (ICIs), such as anti-PD-1 and anti-PD-L1 antibodies, have become a standard option for the management of locally advanced and metastatic lung cancer (1). Approximately 20% of patients with non-small-cell lung cancer (NSCLC) are in stage IIIA (2), with a 3-year overall survival of 30% and no major treatment advances in the past 25 years (3). Recently, chemo-immunotherapy in stage IIIA NSCLC has led to significant pathological responses and downstaging, together with favorable progression-free survival (PFS) and overall survival (OS) at 24 months (4). Also, in the NADIM study, PD-L1 expression was strongly associated with pathologic complete response (pCR), while no significant association was found between PD-L1 tumor proportion score (TPS) and PFS or OS. Besides, in a randomized phase III study, neoadjuvant chemo-immunotherapy has showed significant improvement in pCR rate compared to chemotherapy for resectable (IIIB–IIIA) NSCLC (5). Of note, epidermal growth factor receptor (EGFR) mutations have been generally exclusion criteria in above-mentioned trials and also the IMpower 030 study (6), although patients with EGFR mutations have been allowed in the KEYNOTE-671 study, another neoadjuvant chemo-immunotherapy trial which is still going on (7). Therefore, it is not clear whether a stage IIIA EGFR mutant NSCLC patient with strong PD-L1 expression may benefit from chemo-immunotherapy. Here, we report a case of a stage IIIA EGFR mutant NSCLC with high PD-L1 TPS of 80% yet presented with primary resistance to chemo-immunotherapy, which might be due to rare infiltration of CD8^+^ T cells in the tumor microenvironment.

## Case Description

**Figure 1:**
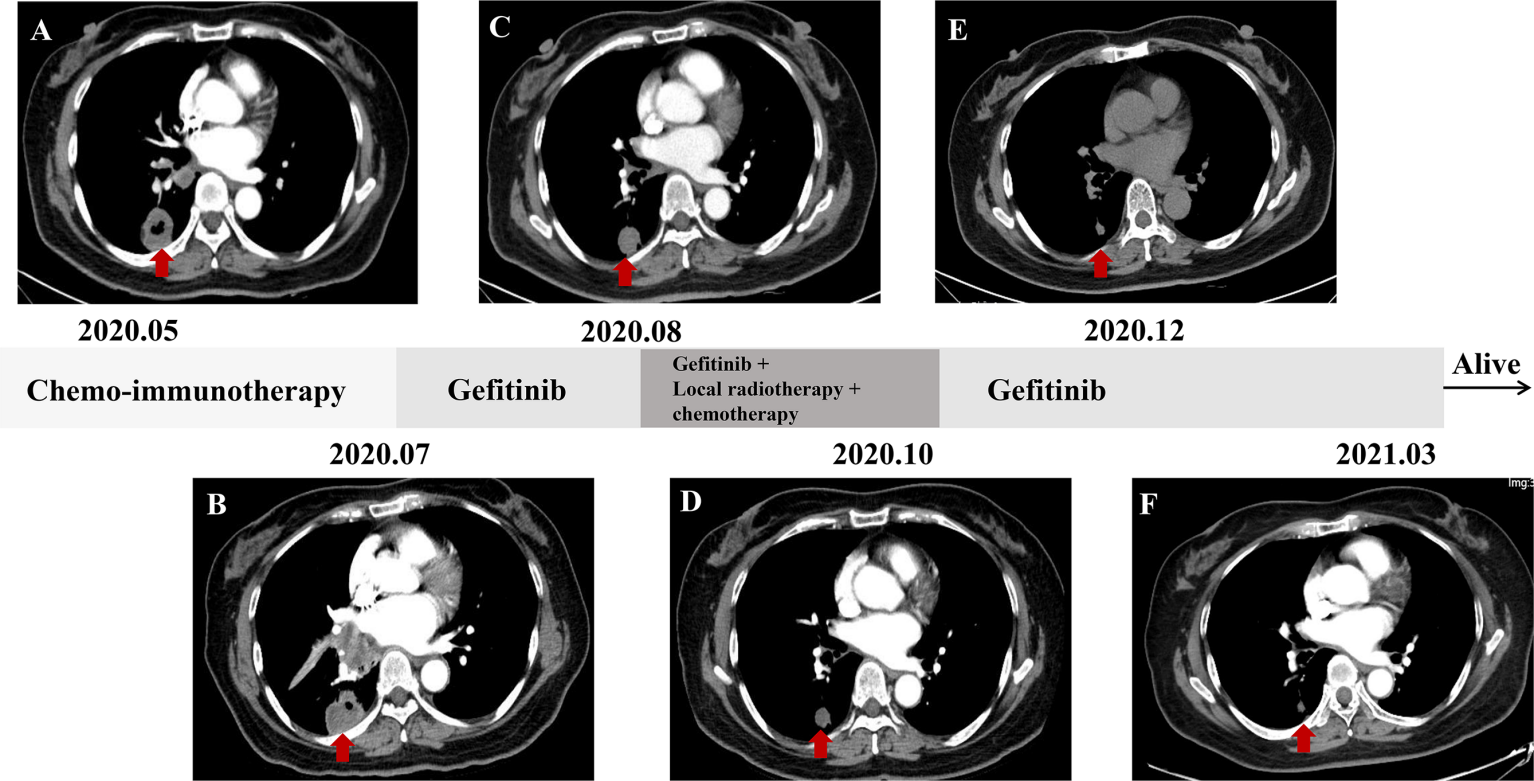
Chest CT scanning of the patient. **(A)** baseline; **(B)** after one cycle of neoadjuvant immuno-chemotherapy (albumin-bound paclitaxel + carboplatin + nivolumab 240 mg); **(C–F)** chest images at indicated time points.

**Figure 2:**
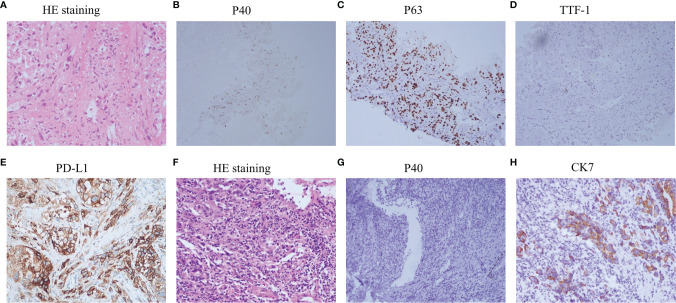
Examinations of pathology and immunohistochemistry. **(A–E)**, Histology of primary lung lesion (CT guided biopsy) and immunohistochemistry analysis, PD-L1 expression; **(F–H)** histology of enlarged lung lesion (re-biopsy through electronic bronchoscopy) and immunohistochemistry analysis.

**Figure 3:**
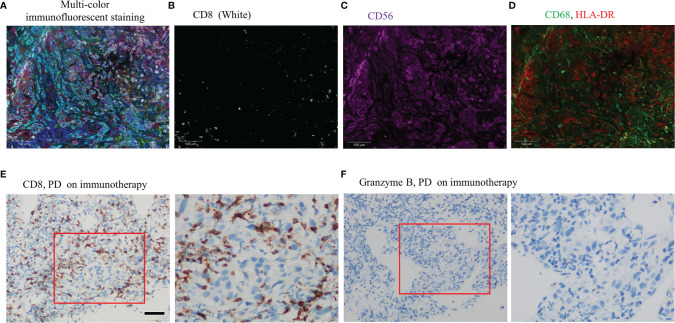
Examinations of tumor microenvironment on tumor tissue sample as indicated. **(A)** Multi-color immunofluorescent staining of tumor microenvironment (white: CD8, purple: CD56, green: CD68, red: HLA-DR, cyan: panCK/S100, blue: DAPI) (magnification ×200); **(B)** CD8 (white) (magnification ×200); **(C)** CD56 (purple) (magnification ×200); **(D)** CD68 (green), HLA-DR (red) (magnification ×200); **(E)** Immunohistochemistry staining of CD8 with tumor tissue on disease progression of albumin-bound paclitaxel + carboplatin + nivolumab, Scale bar 50 um; **(F)** Immunohistochemistry staining of Granzyme B with tumor tissue on disease progression of albumin-bound paclitaxel + carboplatin + nivolumab, Scale bar 50 um.

A 57-year-old nonsmoking female patient was admitted to the Daping Hospital of Army Medical University on May 6, 2020, with cough since 10+ days ago. The physical examination showed no abnormalities. Computed tomography (CT) revealed a mass in the lower lobe of the right lung (3.9 × 3.1 cm) (**Figure 1A**). CT-guided biopsy of the mass followed by the histopathological diagnosis confirmed squamous cell lung carcinoma (**Figures 2A**–**D**). A brain magnetic resonance imaging and a bone single photon emission computed tomography showed no distant metastasis. The patient was therefore diagnosed as T2aN2M0, stage IIIA squamous cell lung carcinoma according to the VIII TNM edition. PD-L1 was found positive (TPS = 80%) using immunohistochemistry (Ventana SP263; **Figure 2E**). After a multidisciplinary team panel discussion, from a “neoadjuvant” perspective, the patient received one cycle of chemo-immunotherapy (albumin-bound paclitaxel + carboplatin + nivolumab 240 mg). There were no severe adverse events after initial treatment. However, the patient suffered from symptoms related to disease progression such as cough, hemoptysis and fever after 3 weeks of treatment. Chest CT illustrated an enlargement of the right lung lesion (4.3×3.1cm) with the obstruction of the right middle bronchus (**Figure 1B**), lung atelectasis and spinal metastases. A re-biopsy through electronic bronchoscopy was performed and a poorly differentiated lung adenocarcinoma was reported in the right middle bronchus tissue by pathologic analysis (**Figures 2F**–**H**). The patient was finally diagnosed as adenosquamous carcinoma. Thereafter, a tissue-based next generation sequencing (NGS, 733 genes, 4.53 Mb, 3D Medicines Inc., China) revealed EGFR p.L858R mutation and TP53 p.R248G mutation. Afterwards, gefitinib was given to the patient (250 mg, once daily) which was well tolerated with slight skin rash that did not require any medical intervention. After one month of treatment, the symptoms of the patient were obviously relieved, and the primary tumor was significantly regressed (**Figure 1C**). Nevertheless, an enlargement of spinal metastases was found and then local radiotherapy combined systemic chemotherapy (albumin-bound paclitaxel + carboplatin; for two cycles) was implemented in addition to gefitinib without any adverse events. Then the spinal lesion was under control and gefitinib alone was still ongoing with persistent regression of primary lung lesion up to Mar 28, 2021 (**Figures 1D**–**F**). We further performed another tissue-based NGS in the baseline sample of squamous cell carcinoma and found the same genetic mutations of EGFR and TP53. Furthermore, a multi-color immunofluorescent staining of pre-treatment tumor sections demonstrated spare infiltration of CD8^+^ T cells (91/mm^2^) and CD68^+^HLA^−^DR^+^ M1 macrophages (21/mm^2^) in the tumor parenchyma, while the infiltration of CD56^dim^ NK cells was high (1,271/mm^2^) (**Figures 3A**–**D**). Further examination of CD8 immunohistochemistry staining on tumor tissue at disease progression on immunotherapy found intense CD8^+^ T cell infiltration (**Figure 3E**), as compared to that of baseline. However, very few of these cells expressed Granzyme B (**Figure 3F**), indicating little cytotoxicity of T cells.

## Discussion

In the current case, we have reported a stage IIIA EGFR mutant NSCLC patient with high PD-L1 expression who showed primary resistance to nivolumab with platinum-based chemotherapy. Further detection of pre-treatment tumor microenvironment revealed rare infiltration of CD8^+^ T cells. Therefore, the case indicated that a comprehensive detection of PD-L1 expression, driver gene status, and tumor immune microenvironment might be helpful for the treatment options.

Several studies have reported that EGFR mutant NSCLC patients benefit little from ICIs, especially in the setting of immuno-monotherapy. A meta-analysis indicated that an ICI as second-line therapy did not improve OS over that with docetaxel therapy in EGFR-mutated advanced NSCLC (8). In a prospective study, among ten EGFR mutant NSCLC patients, first-line pembrolizumab showed no response (seven cases with strong PD-L1 expression) (9). In another study, genomic alterations in EGFR were even suggested to be associated with hyper-progressive disease to ICIs (10). However, the use of ICIs for EGFR mutant NSCLC should not be completely ruled out, since dramatic response to PD-1/PD-L1 inhibitors have been reported in some EGFR mutant cases with strong PD-L1 expression (11). Meanwhile, the combination of chemo-immunotherapy in those patients has been tested in several randomized trials. The IMpower 130 trial failed to improve OS of the outcome of EGFR-mutant NSCLC patients when treated with atezolizumab plus carboplatin and nab-paclitaxel versus chemotherapy alone (12). By contrast, according to the IMpower 150 study, patients with EGFR mutations benefitted from atezolizumab plus bevacizumab, carboplatin, and paclitaxel (ABCP) regimen compared with BCP regimen (13), which suggested that the addition of bevacizumab to chemo-immunotherapy might confer activity to PD-L1 inhibition in EGFR-mutant NSCLC. One possible reason might be that bevacizumab could regulate tumor microenvironment such as promoting T-cell tumor infiltration by normalizing tumor vasculature (14). Other prospective clinical trials such as the KEYNOTE**-**789 and the CheckMate-722 which evaluate the role of chemo-immunotherapy combination in EGFR-mutant advanced NSCLC patients are in expectation (15, 16). However, in a neoadjuvant setting, it is still not clear whether EGFR-TKIs or immunotherapy should be given in the first place to those with EGFR mutations. The Phase III NeoADAURA study will evaluate the efficacy and safety of neoadjuvant osimertinib in patients with resectable EGFR-mutant NSCLC (17). In the current study, we have reported a stage IIIA EGFR mutant NSCLC with high PD-L1 expression, for whom nivolumab plus chemotherapy brought little benefit. Taken together, our case indicated that PD-L1 expression alone might not be enough to predict immunotherapy efficacy. Other aspects, such as tumor microenvironment, may also need to be considered.

Tumor immune microenvironment (TIME), comprising multiple immune cells, has important roles in predicting efficacy of ICIs. For example, infiltration of CD8^+^ T cells can predict benefit from PD-1 inhibitors in lung cancer patients (18). However, mutational activation of EGFR might downregulate MHC-I expression, which could result in a decreased number of infiltrating CD8^+^ T cells, then contributing to the poor response to ICIs (19). Furthermore, high levels of HLA-DR^+^/CD68^+^M1 macrophages are independent prognostic factors of prolonged survival in NSCLC (20). Nevertheless, an analysis demonstrated that a high proportion of CD56^+^CD3^−^ cells was associated with a reduction in the proportion of CD4^+^ tumor-infiltrating lymphocytes (TILs) and, to a greater degree, proportion of CD8^+^ TILs (21). There out, the presence of M1 macrophages or NK cells also play important roles in predicting response cancer immunotherapy (20, 21). In our study, there is a baseline infiltration of CD56 dim NK cells and low CD8^+^ T cells from tumor cells. In addition, the increase of CD8^+^ T cells with low Granzyme B staining on post treatment samples suggests that these CD8^+^ T cells are bystanders rather than tumor specific. Those facts may explain primary resistance of the patient to nivolumab plus chemotherapy even with high PD-L1 expression. Taken together, comprehensive analysis of PD-L1 expression, driver gene status, and TIME may better predict the efficacy of immunotherapy.

Our study has the following limitations. First, the genetic testing was not performed at baseline, which was important for a female patient with no smoking history and small sample tissue. Secondly, the PD-L1 and multi-color immunofluorescent of the re-biopsy tissues after disease progression were not implemented on comparison with that of the baseline tissues due to shortage of samples.

In conclusion, our case suggests a possible biological rationale which could explain the resistance to chemo-immunotherapy, indicating that comprehensive detection of PD-L1 expression, driver gene status, together with tumor immune microenvironment may offer a better prediction of treatment efficacy for EGFR mutant NSCLC patients, even in the case of high PD-L1 expression.

## Data Availability Statement

The original contributions presented in the study are included in the article/supplementary material. Further inquiries can be directed to the corresponding authors.

## Ethics Statement

The studies involving human participants were reviewed and approved by the Daping Hospital, Army Medical University. The patients/participants provided their written informed consent to participate in this study.

## Author Contributions

Concept and design: LL, HY. Acquisition, analysis, or interpretation of data: ZQ, ZX, MQ, LN, WR, and LZ. Drafting of the manuscript: ZQ, ZX, and LL. Critical revision of the manuscript for important intellectual content: All authors. Obtained funding: LL, HY. Supervision: HY. All authors listed have made a substantial, direct, and intellectual contribution to the work and approved it for publication.

## Funding

This work was supported by the Daping Hospital of Army Medical University (2019CXLCA003, 2019CXLCB011) and a Science Foundation for Outstanding Young People of the Army Medical University. The funders of the current study had no role in study design, data collection and analysis, decision to publish, or preparation of the manuscript.

## Conflict of Interest

The authors declare that the research was conducted in the absence of any commercial or financial relationships that could be construed as a potential conflict of interest.

## Publisher’s Note

All claims expressed in this article are solely those of the authors and do not necessarily represent those of their affiliated organizations, or those of the publisher, the editors and the reviewers. Any product that may be evaluated in this article, or claim that may be made by its manufacturer, is not guaranteed or endorsed by the publisher.
